# The Current Role of the Heavy/Light Chain Assay in the Diagnosis, Prognosis and Monitoring of Multiple Myeloma: An Evidence-Based Approach

**DOI:** 10.3390/diagnostics11112020

**Published:** 2021-10-30

**Authors:** Rafael Ríos-Tamayo, Noemí Puig, Macarena Algarín, José Luís García de Veas Silva, Nuno Barbosa, Cristina Encinas, José Ángel Hernández, Rafael Alonso, María Luisa Campos, Teresa Rodríguez, Alberto Leivas, María José Olivares, María José Sánchez, Bruno Paiva, Juan José Lahuerta, Joaquín Martínez-López

**Affiliations:** 1 Hematology Department, Hospital Universitario Virgen de las Nieves, 18014 Granada, Spain; 2 Instituto de Investigación Biosanitaria de Granada (Ibs.GRANADA), 18014 Granada, Spain; mariajose.sanchez.easp@juntadeandalucia.es; 3 Centro de Investigación Biomédica en Red de Epidemiología y Salud Pública (CIBERESP), 28029 Madrid, Spain; 4 Hematology Department, Hospital Universitario de Salamanca (HUSAL), IBSAL, IBMCC (USAL-CSIC) CIBERONC, 37007 Salamanca, Spain; noepuig@gmail.com; 5 Scientific Department, The Binding Site Iberia, 08038 Barcelona, Spain; macarena.algarin@bindingsite.com (M.A.); nuno.barbosa@bindingsite.com (N.B.); luisa.campos@bindingsite.com (M.L.C.); alberto.leivas@bindingsite.com (A.L.); 6 Molecular Diagnosis Laboratory Department, Hospital Universitario Virgen del Rocío, 41013 Sevilla, Spain; Jose6@outlook.com; 7 Hematology Department, Hospital General Universitario Gregorio Marañón, IiGM, 28009 Madrid, Spain; crisenro@hotmail.com; 8 Hematology Department, Hospital Universitario Infanta Leonor, 28031 Madrid, Spain; jahr_jahr2006@yahoo.es; 9 Hematology Department, Hospital Universitario 12 de Octubre, 28041 Madrid, Spain; rafa_alonsofdez@yahoo.es (R.A.); jmarti01@med.ucm.es (J.M.-L.); 10 Immunology Department, Hospital Universitario Virgen de las Nieves, 18014 Granada, Spain; teresa.rodriguez.ruiz.sspa@juntadeandalucia.es (T.R.); mjose.olivares.sspa@juntadeandalucia.es (M.J.O.); 11 Registro de Cáncer de Granada, Escuela Andaluza de Salud Pública (EASP), 18080 Granada, Spain; 12 Department of Preventive Medicine and Public Health, University of Granada, 18016 Granada, Spain; 13 Clínica Universidad de Navarra, CIMA, CIBERONC, IDISNA, 31008 Pamplona, Spain; bpaiva@unav.es; 14 Instituto de Investigación del Hospital Universitario 12 de Octubre, 28041 Madrid, Spain; JJLAHUERTA@telefonica.net

**Keywords:** multiple myeloma (MM), heavy/light chain (HLC) assay, Hevylite^®^, diagnosis, prognosis, monitoring

## Abstract

Despite tremendous progress being made in recent years, multiple myeloma (MM) remains a challenging disease. The laboratory plays a critical role in the overall management of patients. The diagnosis, prognosis, clinical monitoring and evaluation of the response are key moments in the clinical care process. Conventional laboratory methods have been and continue to be the basis of laboratory testing in monoclonal gammopathies, along with the serum free light chain test. However, more accurate methods are needed to achieve new and more stringent clinical goals. The heavy/light chain assay is a relatively new test which can overcome some of the limitations of the conventional methods for the evaluation of intact immunoglobulin MM patients. Here, we report an update of the evidence accumulated in recent years on this method regarding its use in MM.

## 1. Introduction

Multiple myeloma (MM) is a complex and heterogeneous hematological malignancy characterized by the proliferation of clonal plasma cells (PCs) in the bone marrow (BM). The substitution of normal PCs with MM cells and the overproduction of a monoclonal protein (MP) eventually lead to the development of organ damage, summarized in the acronym CRAB (hypercalcemia, renal insufficiency, anemia and bone lesions), and to an immunosuppressed state.

Monoclonal intact immunoglobulins (Igs) and free light chains (FLCs) secreted by malignant PCs are widely considered as biomarkers of tumor burden. International guidelines recommend the detection and quantification of these MPs by using complementary techniques including serum and urine protein electrophoresis (SPEP/UPEP), serum and urine protein immunofixation (sIFE/uIFE) and the serum FLC assay (Freelite; The Binding Site; Birmingham, UK) (Table 1) [1,2,3]. An accurate analysis of this MP is of utmost importance, since it directly impacts the diagnosis, prognosis, and follow-up of patients with MM and other monoclonal gammopathies (MGs).

Regarding the diagnosis of MM, an involved/uninvolved serum FLC ratio ≥100 is considered a biomarker of malignancy, as long as the amount of involved FLC is ≥100 mg/L. This feature, together with the presence of ≥10% clonal PCs in the BM or a biopsy-proven plasmacytoma, is sufficient to diagnose this disease. Additionally, the quantification of the MP is important in order to diagnose premalignant asymptomatic stages that precede MM, including the monoclonal gammopathy of undetermined significance (MGUS) and smoldering MM (SMM) [4,5]. These entities are both characterized by the absence of CRAB. MP levels <30 g/L in serum or <500 mg/24 h in urine and <10% MM cells in BM define MGUS, which has an overall risk of transformation to MM of 1% per year [6]. MP levels ≥30 g/L in serum or ≥500 mg per 24 h in urine and/or 10–60% of MM cells in BM are indicative of SMM, that has an annual risk of progression to active MM of 10% during the first 5 years after diagnosis [7].

The prognosis of patients with these MGs is heterogeneous and, thus, it is important to identify those at higher risk to adapt their clinical management. In MM, several risk stratification models have been developed, such as the Durie–Salmon staging system [8] or the International Stage System (ISS) [9], later revised (R-ISS) [10]. However, only the Durie–Salmon staging system considered the concentration and the isotype of the MP; however, these criteria have been replaced by the ISS. In contrast, in SMM, the MP isotype and its concentration in serum are included in the majority of the widest spread models to identify patients with a higher risk of progression to active MM. For MGUS patients, the Mayo Clinic stratification model contemplates the MP concentration, the MP isotype and the serum FLC ratio as risk factors for progression [11]. Regarding SMM, there is growing evidence supporting the treatment of patients with high risk of progression before they develop symptoms [12,13,14]. Consequently, in recent years, we have witnessed the appearance of various models stratifying the risk of progression, to identify this subgroup of patients. In an attempt at standardization, the IMWG has recently supported the use of the 2/20/20 risk stratification system, which is based on the concentration of the MP, the grade of BM infiltration by MM cells, and the serum FLC ratio [15].

Finally, the evaluation of the response is also based on changes observed on the MP concentration. IFE negative results define complete remission (CR). Different grades of MP decrease measured by SPEP/UPEP determine very good partial responses (VGPRs), partial responses (PRs) or minimal responses. Lastly, increases in MP levels reveal patients who are progressing. In those cases in which the MP concentration is low at baseline (<1 g/dL in serum and <200 mg/24 h-urine), changes in the difference between the involved and the uninvolved FLCs are measured [16].

Therefore, analyzing the MP is crucial in the daily clinical practice to perform an accurate diagnosis, prognosis, and monitoring workup in MGUS, SMM and MM patients. However, conventional techniques are associated with some issues (heterogeneous MP migration, low analytical accuracy for low MP concentrations, interlaboratory variability, etc.) that can negatively impact results [17]. Considering these limitations, new more sensitive, specific, and reliable tests could help to better determine the MP, thus facilitating clinical decisions.

Hevylite^®^ is an alternative fully automatized immunoassay that specifically detects the different heavy/light chain (HLC) pairs (IgGκ/IgGλ, IgAκ/IgAλ, IgMκ/IgMλ), thus providing an accurate quantification of the involved/monoclonal HLC (iHLC) pair and the uninvolved/polyclonal HLC (uHLC) pair of the same isotype. Here, we perform a comprehensive review of the additional information provided by different parameters of the HLC assay (see Table 2) in the diagnosis, prognosis, and monitoring of patients with MGs secreting monoclonal intact Igs. Considering the reported evidence, we will try to define when this assay could complement conventional techniques and help in daily clinical practice.

## 2. Analytical Accuracy and Concordance with Conventional Techniques

Since its introduction by Bradwell et al. [18], different studies have validated the performance of the HLC assay to quantify MPs in serum, showing, in general, a good correlation between iHLC levels and the MP concentration obtained by SPEP [19,20,21,22,23,24,25]. However, some discrepancies between these techniques have been reported. Katzmann et al., despite finding a good correlation between the iHLC and the MP in IgG MM patients, observed that the IgA MP concentration by SPEP was almost 1 g/dL higher than the reported iHLC [20]. Similarly, other studies have informed an inferior grade of correlation between the iHLC and MP levels measured by SPEP in IgA as compared to IgG samples [23,24]. In some of them, the good correlation between the summation of the iHLC and the uHLC concentrations and total IgA measurement by nephelometry or turbidimetry led the authors to hypothesize that this could be due to non-accurate measurements of IgA MPs migrating in the β-region. In this regard, Ludwig et al. found it difficult to monitor 26/56 (46%) IgA and 4/100 (4%) IgG MM patients by SPEP due to the co-migration of the MP with other serum proteins in the β-region [26]. Similar results were observed by Boyle et al., who were not able to quantify the MP in 16 out of the 65 IgA MM patients with β-region M-spikes due to the presence of comigrating bands. In addition, 13 IgA patients with gamma migration M-spikes were also not quantifiable by SPEP in that study [24].

Numerous studies have confirmed the good correlation between the summation of the iHLC and the uHLC concentrations with total immunoglobulin IgG, IgA and IgM levels, demonstrating the accuracy of the assay to quantify mono and polyclonal Igs [18,23,24,26,27,28,29].

Importantly, the limit of detection of the HLC assay is lower than that of conventional methods (SPEP, capillary zone electrophoresis and IFE) currently used for the detection of monoclonal intact Igs in serum (see Table 3).

There is almost no evidence regarding the interference of therapeutic monoclonal antibodies with the HLC assay. Given that the isotype of current approved therapeutic monoclonal antibodies is IgGκ, patients with IgG MM are the most susceptible to such interference. In this respect, Murata et al. supplemented serum samples from two IgGκ MM patients with therapeutic monoclonal antibodies at concentrations similar to the serum maximum concentration values found in MM patients after dose 7 at 16 mg/kg of daratumumab and after dose 4 at 20 mg/kg of elotuzumab. The first sample belonged to a IgGκ MM patient in CR. In this case, although data from non-supplemented serum were not reported, in samples to which daratumumab and elotuzumab were added, a normal HLCr was shown. However, monoclonal bands were visible by both SPEP and sIFE, which could lead one to consider the loss of the CR response. The second sample was from a IgGκ MM patient in <CR. The non-supplemented serum sample exhibited an abnormal HLCr. When daratumumab and elotuzumab were added to the sample, a slight increase in IgGκ levels was observed and the HLCr remained altered. SPEP and sIFE detected the presence of one monoclonal band in the daratumumab-supplemented samples and two bands in the elotuzumab supplemented sample [30]. Further studies will reveal if, as suggested by these results, therapeutic monoclonal antibodies will slightly affect IgGκ concentration, but not enough to alter the HLCr.

## 3. The HLC Ratio as a Biomarker of Clonality

Similar to the serum FLC test, the HLC assay can provide a sensitive measurement of clonality through the HLC ratio (HLCr) in patients secreting monoclonal intact Igs. The global diagnostic sensitivity of the HLCr is close to 99% in MM as shown by different studies detailed in Table 4. Baseline measurements are necessary to interpret the results subsequently obtained during the follow-up.

### 3.1. Prognostic Value

#### 3.1.1. At Diagnosis

Several studies have shown the association of an abnormal HLCr with a worse outcome in newly diagnosed MM (NDMM) patients. Bradwell et al. analyzed a large cohort of 325 NDMM patients and observed that those with an HLCr below or above median values (<0.018 or >93.5 for IgG MM, and <0.01 and >462 for IgA MM) displayed shorter progression-free survival (PFS). However, when IgA NDMM patients were separately analyzed, the prognostic value of the HLCr could not be validated. In spite of this, HLCr <0.01 or >200 and β2-microglobulin (β2M) levels were the only two independent factors associated with PFS on the multivariate analysis. Based on these two parameters, a new stratification model was proposed [19] with greater prognostic value than the ISS.

Similarly, Ludwig et al. also found that an extremely abnormal HLCr, here defined by HLCr <0.022 or >45, was significantly correlated with shorter overall survival (OS) in a cohort of 156 NDMM patients. The multivariate analysis also identified the extremely abnormal HLCr and the β2M concentration as independent risk factors impacting survival [26].

In line with previous results, Koulieris et al. showed that among the 103 NDMM evaluated in their study, those presenting with an iHLC/uHLC ratio higher than the median values (21.5 for IgG and 72.4 for IgA) exhibited shorter time to treatment. Interestingly, among the different disease variables analyzed, only the β2M concentration, a highly abnormal iHLC/uHLCr and platelet counts remained significant as independent risk factors for OS in the multivariate analysis [31].

Finally, a Spanish group evaluated the prognostic value of the HLCr in 183 NDMM patients from three different clinical trials (GEM2005MENOS65, GEM2005MAS65, GEM2010MAS65) including transplant-eligible and non-eligible patients. According to previous studies, they found a statistically significant shorter PFS for patients with a baseline highly abnormal HLCr <0.29 or >73, independently of the isotype. This finding was confirmed in the multivariate analysis, in which the highly abnormal HLCr was among the factors significantly associated with increased risk of progression [21].

#### 3.1.2. At Follow-Up

The degree of alteration of the HLCr has been related to the response achieved in treated MM patients. At the time of maximum response, Ludwig et al. observed a correlation between the HLCr and the response achieved. The lower the iHLC/uHLC ratio, the deeper the response reached [26]. Similar results were found by Batinic et al. in a subset of 90 unselected MM patients at different stages of the disease, in which 100% of patients in PR, 59% in VGPR and 22% in CR showed an abnormal HLCr [37]. In line with those results, the aforementioned Spanish group reported 100%, 100%, 88%, 66% and 48% of patients with abnormal HLCr in progressive disease (PD), stable disease (SD), PR, VGPR and CR in their study [21]. Lastly, in the study performed by Suehara et al., the following percentages of normal HLCr were found among different response categories for IgA and IgG MM patients: 0% and 13% in PR, 28% and 64% in VGPR, 92% and 87% in CR and 91% and 84% in stringent CR, respectively [33].

Regarding the prognostic value of the HLCr at maximum response, different results have been published. Ludwig et al. observed that the normalization of the HLCr at the time of maximal response was correlated with a better outcome among patients in ≥PR, but not in those in ≥VGPR [26]. However, only 36 patients in ≥VGPR were evaluated in that analysis. When Drayson et al. evaluated 120 IgA MM patients from the MRC IX trial achieving ≥VGPR at maximum response or three months after transplant, a statistically significant association between the HLCr normalization and a longer PFS was observed [38]. In accordance with Drayson et al., the survival analysis of 85 IgA MM patients achieving ≥VGPR at best response reported by Suehara et al. revealed that patients normalizing the HLCr had longer OS. However, when survival analyses were performed according to the MP isotype, a significant benefit in terms of OS was observed for IgA, but not IgG, MM patients. This result seems to suggest that normalization of the HLCr may impact differently on IgG or IgA MM patients [33].

Additionally, several published clinical cases suggest that the HLCr may add relevant information during the monitorization of MM patients. Bradwell et al. observed that in three out of the five evaluated IgG MM patients in CR, the alteration of the HLCr was able to detect the subsequent relapse earlier than SPEP/IFE [18]. In agreement with those results, Espiño and colleagues found that the iHLC/uHLC ratio was the most accurate factor to predict progression in 11 IgG MM patients, being able to detect progression with 4.5 months of anticipation [39]. Another case was reported by Ludwig et al., in which the HLCr was the first indicator of the relapse. However, this MM patient relapsed only secreting monoclonal intact Igs, without showing abnormal serum FLC ratio [26]. This observation suggests that it could be interesting to monitor both HLC and FLC parameters to follow the evolution of clones secreting different types of MPs, as suggested by Gagliardi and colleagues [40]. Additionally, Chae et al. observed an alteration of the HLCr in two patients in CR due to decreased uHLC levels. IFE turned positive 1.5 and 3 months after the HLCr changed from normal to abnormal in these patients [22].

## 4. dHLC and HLCr for the Evaluation of the Response to the Treatment

Two major studies have focused on the HLC assay’s ability to categorize the level of response to therapy.

Michallet and colleagues assessed the response to therapy in 509 NDMM from the IFM 2009 trial, comparing standard IMWG response criteria [16] with the subsequently described HLC criteria. PD was defined by increases of >25% in the difference between the iHLC and the uHLC (dHLC), SD by a decrease inferior to 50%, PR by a decrease between 50 and 89%, and VGPR by a decrease ≥90%. CR was defined by BM infiltration <5%, the absence of plasmacytomas and a normal HLCr. At the end of consolidation, there was a moderate agreement between responses categorized by either SPEP/sIFE or dHLC/HLCr. Notably, the agreement rate was poorer within patients classified in VGPR according to the IMWG criteria, since almost half of these patients were classified in CR by the HLC assay. More importantly, this subgroup of patients considered in CR by HLC exhibited a longer PFS than those classified by both methods in VGPR. Similar findings were found in the population achieving conventional CR. Within that group, a subset of patients with abnormal HLCr, thus classified in VGPR by the HLC criteria, was significantly associated with a worse outcome. These results suggest that response assessment based on the HLC assay was more accurate as compared to the standard IMWG response criteria. Indeed, analysis of minimal residual disease (MRD) at a sensitivity of 10^−4^ in BM supported the HLC results. After consolidation, 62% of patients were IFE-positive, as compared to the 37% with an abnormal HLCr and the 34% which were MRD-positive. Moreover, MRD positivity was associated with shorter PFS in patients classified in both VGPR and in ≥CR according to IMWG criteria. In contrast, MRD positivity was only associated with a worse outcome in patients with normalized HLCr and, therefore, in CR by HLC criteria, but not in those in VGPR exhibiting abnormal HLCr [34]. Altogether, these observations suggest a role for the HLC assay as a serum marker for making decisions on the appropriate timing for MRD BM testing.

A different approach was followed by Fouquet and colleagues, who based HLC response assessment on changes in the HLCr. CR was defined by a normal HLCr, VGPR by a >94% decrease on the HLCr, PR by a decrease between 60% and 94%, SD between 24% and 60% and PD by an increase of >24% with respect to the baseline HLCr. A good agreement with standard response criteria was obtained when patients were divided in ≥PR and <PR. Survival analysis performed according to the HLC criteria revealed a benefit in terms of OS for patients achieving ≥PR as defined by the HLCr [25].

## 5. uHLC: Polyclonal Non-Tumoral Isotype-Specific Immunoglobulin Suppression

Classical immunoparesis of Igs unrelated to the tumor isotype has been generally associated with poorer outcomes in MGs [41,42,43,44,45,46,47,48,49,50,51,52]. In line with those reports, increasing evidence regarding the prognostic value of the non-tumoral/uninvolved Ig associated to the tumoral isotype, but also of the alternative light chain (e.g., the IgGκ in a IgGλ secreting tumor), has been published (see Table 5). This phenomenon has been termed isotype-matched immunoparesis (IMI).

### 5.1. Prognostic Value

#### 5.1.1. At Diagnosis

Approximately one third of the patients with MGUS shown IMI in a large study evaluating 999 individuals in this premalignant stage [32]. This proportion was higher (41%) in the cohort evaluated by Magnano et al., although almost one quarter of the patients included in this study had SMM and were, therefore, in a more advanced stage of the disease [54]. In this regard, Jimenez and colleagues reported that half of the 307 evaluable MGUS samples shown IMI, although the percentage of low-risk MGUS in this cohort was 28%, as compared to the 48% found in the study performed by Katzmann et al. Of note, they observed significantly lower levels of IgG uHLC as the risk of progression of MGUS subgroups increased. This finding did not reach statistical significance in IgA and IgM MGUS, probably due to the very low number of patients conforming some of the subsets. More importantly, as a result of a multivariate analysis, severe IMI, contrary to severe classical immunoparesis, was found to be an independent risk factor of progression from MGUS to symptomatic disease [53]. Similar results were obtained by Katzman and colleagues with IMI. Indeed, the incorporation of the IMI as an additional adverse factor to the widely used Mayo Clinic risk stratification system significantly increased the discriminatory power of the model to identify high-risk MGUS patients [32]. In addition, Espiño et al. found that in IgG MGUS patients, the frequency of iHLC/uHLC ratio above 9.5 correlated with the classification of evolving/non-evolving MGUS proposed by Rosiñol et al. [55]. All patients that progressed to MM during the study had an iHLC/uHLC ratio above 9.5 at MGUS diagnosis [39].

Isola et al. prospectively monitored 53 patients with SMM. Severe IMI was found in 51% patients at diagnosis versus the 36% with severe classical immunoparesis. Nine out of the 12 SMM patients that progressed to symptomatic disease after a median follow-up of 2.5 years shown severe IMI at diagnosis [49].

In MM, Bradwell and colleagues, for the first time, analyzed the prognostic value of IMI in a cohort of 339 NDMM patients. They observed that deep IMI was significantly associated with adverse PFS [19]. Similar results were observed by Geng et al. in a cohort of 287 NDMM patients [48]. Accordingly, severe IMI was identified in 85 (54.5%) of the 156 NDMM patients evaluated by Ludwig et al. These patients significantly exhibited shorter OS than those with moderate or without IMI. However, when the 56 IgA MM patients included in the study were separately analyzed, the prognostic value of IMI was not validated [43]. By contrast, Boyle et al. reported severe IMI to be significantly associated with a shorter OS in their study limited to 157 IgA NDMM patients. Contrary results regarding the role of IMI as prognostic factor were observed by Lopez-Anglada et al. in their cohort of 183 NDMM patients, in which severe IMI did not impact neither PFS or OS [21]. Regarding comparisons with classical immunoparesis, Kourelis et al. found both classical immunoparesis and IMI to be significantly associated with a shorter time to treatment in their cohort of 103 NDMM patients [31].

Focusing on the role of uHLC levels as the predictor of infections occurring in the first months after initiating therapy, our group recently found that, contrary to classical immunoparesis, severe IMI was significantly associated to blood stream infections. In contrast, a significant association with severe classical immunoparesis at baseline was not observed. In addition, uHLC values 95% inferior to the lower limit of the normal range (extreme IMI) were significantly associated to early mortality [36].

#### 5.1.2. At Follow-Up

In the GEM-CESAR trial, one of the first curative strategies designed for high-risk SMM patients, IMI was identified in 13 out of the 62 evaluable patients, being this severe in 10 of them at the end of consolidation. In the future, we will know whether these patients are associated with a higher risk of progression [35].

Harutyunyan and colleagues evaluated IMI associated with the response (CR, VGPR, PR, SD and PD) at the time of blood sampling in 189 heterogeneously treated MM patients. They observed that the better the response, the lower the percentage of patients with IMI. In addition, IMI was found to be significantly correlated with a shortened PFS in this cohort of patients [56]. Concordant results obtained at the end of consolidation were reported by Michallet et al., both in all patients and only in those in conventional ≥CR [34], and by Batinic and colleagues in their cohort of 90 unselected MM patients at different stages of the disease [37].

As observed at diagnosis, the opposite results were reported by Lopez-Anglada et al. In their cohort of 89 heterogeneously treated MM patients, severe IMI did not impact neither PFS or OS [21]. In agreement with Lopez-Anglada et al., no significant differences in terms of OS were observed between patients with or without IMI by Suehara and colleagues. Nevertheless, survival analyses were performed only for MM patients in ≥VGPR in this study [33].

## 6. Discussion

Regarding the introduction of the HLC assay in the last published guidelines in 2016, the IMWG stated that more data should be collected to draw conclusions about the role of this assay for intact Igs MM patients [16]. Here, we have carried out an updated review of the most relevant studies evaluating the role of the HLC assay in the diagnosis, prognosis and follow-up of MGUS, SMM and MM in an attempt to define when this assay may provide additional information of interest for the clinical management of these patients.

In spite of analyzing published data according to the different HLC parameters, it is important to remark that all these variables are strongly linked. For instance, the HLCr directly depends on the iHLC and the uHLC concentrations. Thus, the HLCr is influenced by IMI. In addition, although information about all HLC parameters is obtained in each measurement, only some of them have been demonstrated to be clinically relevant in each situation.

In asymptomatic patients, IMI seems to have an interesting role in identifying those patients with higher risk of progression. In MGUS, two large studies have found IMI to be an independent risk factor of progression [32,53]. In fact, the addition of the IMI to the stratification model developed by the Mayo Clinic resulted in the better identification of those patients at highest risk of progression. Moreover, a higher association of IMI rather than classical immunoparesis with progression is reported. Likely, the iStopMM study (NCT03327597), conducting a population-based screening for MGUS in more than 80,000 participants [57], will help to definitively define the role of the HLC assay in this patient population. In SMM, only one prospective study has been reported as suggesting a higher correlation of the risk of progression with IMI than with classical immunoparesis [49]. However, this aspect needs to be validated by other larger prospective studies such as the one that is being implemented in the context of the GEM-CESAR trial. A similar role to that observed for IMI had been previously seen for classical immunoparesis. Whether IMI is a better biomarker than classical immunoparesis will have to be determined by future comparative studies.

Concerning the MM diagnostic workup, although the HLC assay is not yet included in current IMWG diagnostic guidelines, its use could add value at this moment, especially in patients secreting IgA MPs, since HLC measurement is independent of the MP electrophoretic migration pattern [18,23,24,26]. Additionally, several studies illustrated in Table 3 supported the high diagnostic performance of the HLC assay at diagnosis in IgA, IgG and IgM MM patients.

Once the diagnosis is confirmed, a prognostic estimation should be performed. However, some risk stratification systems cannot accurately predict the outcome in a particular MM patient, since some of the considered risk factors are not routinely measured. The HLCr has demonstrated its prognostic value in intact Ig NDMM patients in several studies [19,21,26,31]. Moreover, the HLCr has been identified as a significant independent parameter impacting survival in various multivariate analyses, including well-accepted variables such as β2M, albumin or chromosomal abnormalities. Therefore, the HLCr may help in the real-world setting to stratifying the risk of MM patients at diagnosis. In addition, performing the HLC assay at baseline is necessary to assess future measurements.

This is the case in response assessment. The HLCr has been associated with the response in several studies [26,33,37]. The comparative study between the IMWG criteria based on SPEP/IFE results and the HLC criteria based on dHLC/HLCr performed by Michallet et al. suggests that the HLC assay would allow a more accurate evaluation of the response, especially among patients in conventional VGPR and CR. The better correlation of HLC as compared to IFE with MRD results supported those findings [34]. This estimate seems reasonable, since the limit of detection of the HLC assay for the detection of intact Igs is lower than the exhibited by conventional electrophoretic methods (Table 3). Regarding the best parameter to evaluate the response, it has to be considered that treatment can intensify the grade of immunosuppression in MM patients, and concentrations below the normal ranges of both the iHLC and the uHLC can be frequently observed after therapy. In this scenario, even inverted abnormal HLCr can be observed. For this reason, perhaps, following changes on the dHLC may be more appropriate than variations on the HLCr, as performed by Fouquet and colleagues.

Regarding the monitoring of patients with MM, the evolution of the MP in serum must be followed by SPEP and sIFE (Table 1). With respect to the HLC assay, there are not many studies analyzing sequential samples once consolidation treatment is ended. However, in some clinical cases here collected, the alteration of the HLCr was observed prior to the appearance of the MP by SPEP/IFE due to the decrease in the uHLC concentration that precedes the iHLC expansion [22,26]. These findings suggest an important role of IMI as an early biomarker of relapse. Therefore, observing the evolution of the uHLC in every visit (along with the corresponding HLCr or dHLC in patients in <CR) could lead one to detect relapses earlier than with conventional electrophoretic methods. Confirming these findings in larger cohorts of patients is of utmost importance, since an earlier detection of relapses, followed by an earlier therapeutic intervention, could avoid clinical complications.

Finally, early mortality remains a matter of concern in MM patients. In our population-based registry, infection was the main cause of death at 6 and 12 months from diagnosis, at 38% and 42.9%, respectively [58]. In addition to the infectious risk associated to treatment, patients with MM present a complex dysfunction of the immune system at the cellular and humoral level, which seriously predisposes to bacterial, viral, and fungal infections. In a very recent real-world study including 115 NDMM patients, 21% of the patients suffered a blood stream infection in the first 6 months after initiating therapy, of which 58% died. Furthermore, it was demonstrated for the first time that, contrary to classical immunoparesis, severe IMI was an independent risk factor for blood stream infections and early death in NDMM [36]. These results need to be validated in other independent cohorts, but they suggest that the HLC assay may have a role in the selection of patients requiring preventive therapy to avoid infections.

**Figure 1:**
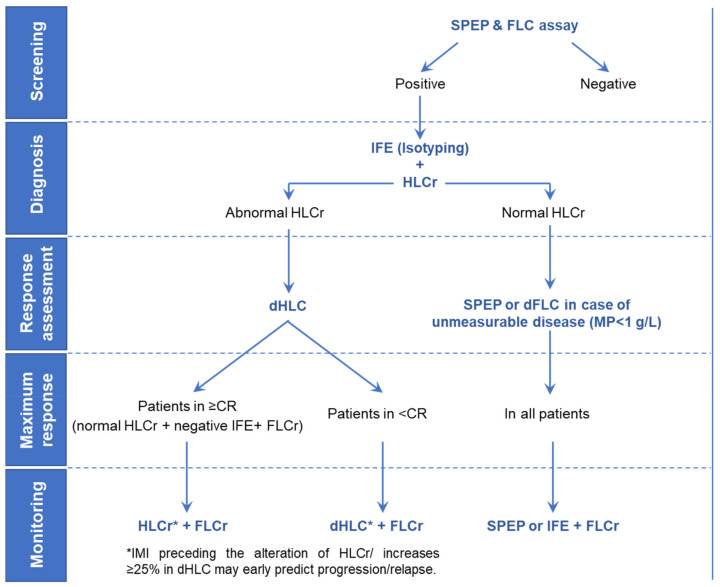
Proposed serological algorithm for the management of the monoclonal protein in patients with multiple myeloma.

In conclusion, to date, no single laboratory test can confidently be used to obtain all the necessary information for the diagnosis, prognosis, monitoring and response evaluation in MM. The HLC assay is a validated, automated, and easy to use method for the MP quantification that overcomes several well-known technical limitations associated to conventional electrophoretic methods. For this reason, it was proposed that the HLC assay could substitute all the current assays in IgA MM patients (SPEP, sIFE and total IgA) with a β-migrating MP. Similar benefits were also described for patients with an IgG and IgM MP migrating in the β-region [20]. If futures studies confirm that HLC response criteria correlate better with the depth of response than the current IMWG criteria, sIFE could be used only at diagnosis to classify the MP. Thereby, when the presence of a MP was detected by SPEP and/or the serum FLC assay in the screening, sIFE could be performed for typing. Once the isotype is identified, the HLC assay could be performed. If the HLCr is normal, as expected, for example, for patients with Bence Jones MM, the evaluation of the response would be performed, attending to SPEP results or dFLC in the case of unmeasurable disease (MP < 1 g/L) following the current IMWG criteria. At the end of treatment, the follow-up would be performed by SPEP or sIFE and the serum FLC assay. In contrast, if the HLCr is altered at baseline, response assessment could be based on the HLC response criteria. sIFE could be performed to confirm the CR in patients normalizing the HLCr. Finally, monitoring could be based on both HLC and FLC results. The combined use of the corresponding assay (HLC or SPEP/sIFE) with the FLC assay ensures the detection of changes on the MP isotype due to clonal evolution [59]. This proposed algorithm to manage the MP (Figure 1) would allow an almost fully automated follow-up of the MP, improving the laboratory workflow and optimizing the laboratory staff time and associated costs.

**Figure 2:**
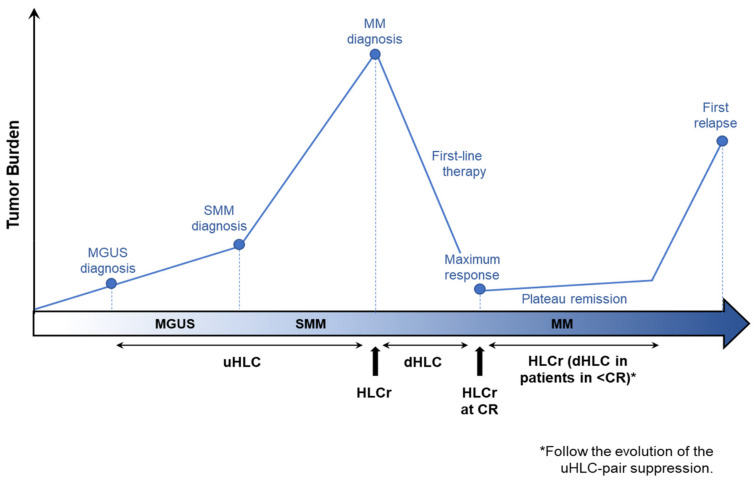
Heavy/light chain parameters providing most relevant information throughout disease.

Moreover, it provides additional information that could help to improve the clinical decision-making. Based on evidence here collected, we consider that the HLC assay may complement SPEP, sIFE and the serum FLC assay, especially in the settings indicated in Figure 2, and that it is ready for prime time.

## Figures and Tables

**Table 1:** Basic laboratory testing in multiple myeloma.

Test	Diagnosis	Prognosis	Monitoring	Response
**CBC**	M	M	M	M
**Blood smear**	M	M	O	M
**SPEP**	M	M	M	M
**sIFE**	M	M	M	M
**UPEP**	M	M	M	M
**uIFE**	M	M	M	M
**sFLC**	M	M	M	M
**Ig**	M	M	M	M
**Renal and liver**	M	M	M	M
**Calcium**	M	M	M	M
**LDH**	M	M	M	M
**Alb and β2M**	M	M	O	NR
**BM a/b**	M	M	O	M
**BM NGF/NGS**	M	M	O	M
**BM FISH**	M	M	O	M

Alb: albumin; β2m: β2-microglobulin; BM a/b: bone marrow aspirate/biopsy; CBC: complete blood count; FISH: fluorescence in situ hybridization [del17p, t(4;14), t(14;16), ampl 1q/gain 1q, del1p, t(11;14)]; sFLC: serum free light chains; Ig: serum immunoglobulin levels; LDH: lactate dehydrogenase; M: mandatory; NGF: next generation flow; NGS: next generation sequencing; NR: not required; O: optional; sIFE: serum immunofixation electrophoresis; SPEP: serum protein electrophoresis; uIFE: urine immunofixation electrophoresis; UPEP: urine protein electrophoresis.

**Table 2:** Description of the heavy/light chain (HLC) assay parameters.

Parameter	Definition	Description	Example in an IgAκ Patient
**iHLC**	Involved HLC	Monoclonal HLC pair produced by MM cells	IgAκ
**HLCr**	κ/λ HLC ratio	Indicates clonality	IgAκ/IgAλ
**dHLC**	Difference HLC	Difference in concentration between the iHLC and the uHLC	IgAκ-IgAλ
**uHLC**	uninvolved HLC	Polyclonal HLC pair of the same isotype. When it is below the normal reference interval (IMI) ^1^	IgAλ

^1^ IMI: isotype-matched immunoparesis.

**Table 3:** Limit of detection of methods analyzing the monoclonal protein in serum.

Method ^†^		Analytical Sensitivity
**SPEP**		1 g/L
**Capillary Zone Electrophoresis**		0.25 g/L
**IFE**		0.1 g/L
**Hevylite^®^**		
	IgGκ	0.115 g/L *
	IgGλ	0.075 g/L *
	IgAκ	0.018 g/L *
	IgAλ	0.016 g/L *
	IgMκ	0.020 g/L *
	IgMλ	0.018 g/L *
**Freelite^®^**		
	κ	0.0006 g/L *
	λ	0.0013 g/L *

† Based on manufacturer’s information and published studies. Adapted from AR Bradwell. 2015. Serum Free Light Chain Analysis plus Hevylite. 7th Edition, The Binding Site Group Ltd., Birmingham. UK. * Lower limit of the measuring range on the Optilite analyzer.

**Table 4:** Heavy/light chain ratio (HLCr) sensitivity displayed at diagnosis in different studies.

Reference	No. of Patients	% of Patients with Abnormal HLCr
[18]	51 MM (18 IgG; 33 IgA)	100%
[31]	103 MM (78 IgG; 25 IgA)	100%
[26]	156 MM (100 IgG; 56 IgA)	100%
[32]	999 MGUS (726 IgG; 117 IgA; 156 IgM)	66%
[19]	339 MM (245 IgG; 94 IgA)	100%
[24]	157 IgA MM	100%
[20]	518 MM (365 IgG; 153 IgA)	97%
[33]	97 MM (61 IgG; 36 IgA)	100%
[34]	509 MM (393 IgG; 116 IgA)	99%
[21]	183 MM	98%
[22]	36 MM (20 IgG; 16 IgA)	100%
[35]	75 SMM (HR) (50 IgG; 25 IgA)	98% IgG; 100% IgA
[36]	115 MM (76 IgG, 39 IgA)	100%

HR: high risk; MGUS: monoclonal gammopathy of undetermined significance; NDMM: multiple myeloma; SMM: smoldering multiple myeloma.

**Table 5:** Prognostic value of baseline isotype-matched immunoparesis (IMI) and/or classical immunoparesis (CIP) observed in different studies.

Reference	Type of IP	Type of MG	n	% with IMI/CIP	Outcome
[41]	CIP	SMM	93	66	TTP
[31]	IMI CIP	NDMM	103	81 74	TTT TTT
[19]	IMI: the lower 2/3rds	NDMM	325	64	PFS
[42]	CIP	NDMM	1755	87	OS
[24]	IMI IMI<50% CIP	NDMM	157	80 33 84	- OS -
[43]	IMI<50%	NDMM RRMM	156 47	54.5 61.7	OS OS
[50]	CIP CIP<25%	NDMM	2558	90 81	OS: ns PFS
[44]	CIP	NDMM	5826	85	OS and PFS
[21]	IMI	NDMM	183	-	PFS and OS: ns
[53]	CIP<50% IMI<50%	MGUS	154	7 14	TTP: ns TTP
[45]	CIP	NDMM	147	84	OS: ns PFS
[46]	CIP	SMM	527	54.3	TTP
[47]	CIP CIP<50%	RRMM(FR)	258	95	PFS. OS: ns OS and PFS
[51]	CIP	NDMM	262	78	OS and PFS
[48]	IMI IMI<50%	NDMM	287	92.3 70	- OS and PFS
[49]	IMI IMI<50% CIP CIP<50%	SMM	53	79 51 54 36	- TTP - -
[36]	CIP<50% IMI<50% IMI<95%	NDMM	115	46 64 18	BSI: ns BSI EM
[52]	CIP	NDMM SMM	242 22	81.4 63.6	OS: ns -

BSI: bloodstream infections; CIP: classical immunoparesis; EM: early mortality; FR: first relapse; HR: high risk; IMI: isotype-matched immunoparesis; IP: immunoparesis; MMS: smoldering multiple myeloma; NDMM: newly diagnosed multiple myeloma; ns: not significant; OS: overall survival; PDR: progression disease risk; PFS: progression-free survival; RRMM: relapsed/refractory multiple myeloma; TTP: time to progression; TTT: time to treatment.

## Data Availability

Not applicable.
